# Supplementary material for: Learning action-oriented models through active inference
Source: PLoS Comput Biol. 2020 Apr 23;16(4):e1007805. doi: 10.1371/journal.pcbi.1007805 (PMC7200021; doi:10.1371/journal.pcbi.1007805)
Supplement: S1 Appendix — In this appendix, we provide derivations for three arrangements of the free energy functional. (PDF) [file pcbi.1007805.s001.pdf]

Appendix 1

In this appendix, we provide three rearrangements of the free energy functional $\mathcal{F}(\phi, o)$, thus providing an intuition as to what the minimization of free energy entails. By defining free energy $\mathcal{F}(\phi, o)$ as the KL-divergence between an approximate posterior $Q(x|\phi)$ and a generative model $P(x, o)$, we can write:

$$\begin{aligned}\mathcal{F}(\phi, o) &= \mathbb{KL}[Q(x|\phi)||P(x, o)] \\ &= \mathbb{E}_{Q(x|\phi)}[\ln Q(x|\phi) - \ln P(x, o)]\end{aligned} \tag{1}$$

where the second equality is simply the definition of the KL-divergence, $\mathbb{KL}[Q||P] = \mathbb{E}_Q[\ln Q - \ln P]$. We can factorize the generative model $P(x, o)$ as $P(x|o)P(o)$, allowing us to rewrite equation 1 as:

$$\begin{aligned}\mathcal{F}(\phi, o) &= \mathbb{E}_{Q(x|\phi)}[\ln Q(x|\phi) - \ln P(x|o)] - \ln P(o) \\ &= \mathbb{KL}[Q(x|\phi)||P(x|o)] - \ln P(o)\end{aligned} \tag{2}$$

Note that the negative log-likelihood of observations $-\ln P(o)$ remains outside of the expectation in the first equality as $P(o)$ does not depend on $Q(x|\phi)$. The second equality demonstrates that free energy can be expressed as the KL-divergence between the approximate posterior $Q(x|\phi)$ and the true posterior distribution $P(x|o)$, minus the log-likelihood of observations $\ln P(o)$. As the KL-divergence is a strictly non-negative quantity, free energy will always be greater than or equal to the negative log-likelihood of observations, $\mathcal{F}(\phi, o) \geq -\ln P(o)$. This means that free energy will be equal to to the negative log-likelihood of observations when the posterior divergence term is equal to zero. Therefore, free energy is an *upper bound* on the negative log-likelihood of observations, a quantity sometimes referred to as *surprisal*. Minimizing free energy will, therefore, minimize surprisal, or equivalently, maximize Bayesian model evidence $P(o)$.

An alternative expression of free energy can be derived through an alternative factorization of the generative model, $P(x, o) = P(o|x)P(x)$, allowing us to rewrite equation 1 as:

$$\begin{aligned}\mathcal{F}(\phi, o) &= \mathbb{E}_{Q(x|\phi)}[\ln Q(x|\phi) - \ln P(o|x) - \ln P(x)] \\ &= \mathbb{E}_{Q(x|\phi)}[\ln Q(x|\phi) - \ln P(x)] - \mathbb{E}_{Q(x|\phi)}[\ln P(o|x)] \\ &= \mathbb{KL}[Q(x|\phi)||P(x)] - \mathbb{E}_{Q(x|\phi)}[\ln P(o|x)]\end{aligned} \tag{3}$$

The final equality of equation 3 demonstrates that free energy can be expressed as the KL-divergence between the approximate posterior $Q(x|\phi)$ and the prior probability of unknown variables $P(x)$, minus the conditional log-probability of observations $P(o|x)$ expected under the approximate posterior. The first of these terms quantifies the *complexity* of the approximate posterior, as it measures how much the approximate posterior changed in order to account for some new observations (i.e. in going from prior to approximately posterior beliefs). The second term measures the *accuracy* of the approximate posterior, as it quantifies how likely the observations are, given the beliefs encoded by the approximate posterior. Therefore, minimizing free energy entails a trade-off between minimizing the complexity of the beliefs encoded by the approximate posterior and maximizing the accuracy of those beliefs.

Finally, we can rearrange free energy as:

$$\begin{aligned}\mathcal{F}(\phi, o) &= \mathbb{E}_{Q(x|\phi)}[\ln Q(x|\phi)] - \mathbb{E}_{Q(x|\phi)}[\ln P(x, o)] \\ &= -\mathbf{H}[Q(x|\phi)] - \mathbb{E}_{Q(x|\phi)}[\ln P(x, o)]\end{aligned} \tag{4}$$

where $\mathbf{H}[Q(x|\phi)]$ is the Shannon entropy of the approximate posterior, defined as $-\mathbb{E}_{Q(x|\phi)}[\ln Q(x|\phi)]$. The final equality demonstrates that minimizing free energy entails maximizing the entropy of the approximate posterior, while also maximizing the expected energy $\mathbb{E}_{Q(x|\phi)}[\ln P(x, o)]$. Maximizing the entropy of the approximate posterior ensures that the approximate posterior provides a generic and parsimonious explanation of the observed data, thereby ensuring that those explanations are not based on highly-specific (i.e. low-entropy) beliefs.
