# Supplementary material for: Learning action-oriented models through active inference
Source: PLoS Comput Biol. 2020 Apr 23;16(4):e1007805. doi: 10.1371/journal.pcbi.1007805 (PMC7200021; doi:10.1371/journal.pcbi.1007805)
Supplement: S2 Appendix — In this appendix, we formally describe the relationship between free energy and expected free energy. (PDF) [file pcbi.1007805.s002.pdf]

Appendix 2

In what follows, we formally describe the relationship between free energy $\mathcal{F}(\phi, o)$ and expected free energy $\mathbf{G}_\tau(\phi_\tau, u_t)$. To help clarify this relationship, we rewrite free energy as $\mathcal{F}_t(\phi_t, o_t)$, where subscript x_t implies the value at time t, thus making explicit the fact that free energy corresponds to the current time t, and where (following Appendix 1):

$$\mathcal{F}_t(\phi_t, o_t) = \mathbb{E}_{Q(x_t|\phi_t)}[\ln Q(x_t|\phi_t) - \ln P(x_t, o_t)] \tag{1}$$

Expected free energy $\mathbf{G}_\tau(\phi_\tau, u_t)$ differs from the free energy functional in equation 1 in three respects. First, it looks to quantify the free energy that is expected to occur at some future time τ. We can attempt to define the free energy for time τ as:

$$\mathcal{F}_\tau(\phi_\tau, o_\tau) = \mathbb{E}_{Q(x_\tau|\phi_\tau)}[\ln Q(x_\tau|\phi_\tau) - \ln P(o_\tau, x_\tau)] \tag{2}$$

However, equation 2 poses a problem. The free energy at the current time point $\mathcal{F}_t(\phi_t, o_t)$ is a *function* of observations o_t because the observations at time t are known. In contrast, the observations at time τ are unknown, meaning that the free energy at time τ cannot be a function of (unobserved) observations. Instead, *beliefs* over observations at time τ are required to evaluate free energy at the future time point τ. By assuming that observations o_τ depend on the unknown variables x_τ (which will be formalized once the generative model has been defined), we can introduce a distribution (or beliefs) over future observations $Q(o_\tau|x_\tau, \phi_\tau)$. Free energy at future time τ can then be evaluated under the expectation of this distribution:

$$\begin{aligned}\mathcal{F}_\tau(\phi_\tau) &= \mathbb{E}_{Q(x_\tau|\phi_\tau)}\Big[\mathbb{E}_{Q(o_\tau|x_\tau,\phi_\tau)}[\ln Q(x_\tau|\phi_\tau) - \ln P(x_\tau, o_\tau)]\Big] \\ &= \mathbb{E}_{Q(o_\tau,x_\tau|\phi_\tau)}\big[\ln Q(x_\tau|\phi_\tau) - \ln P(x_\tau, o_\tau)\big]\end{aligned} \tag{3}$$

Finally, we note that the functional purpose of expected free energy is to quantify the free energy that is expected to occur at time τ *given* the execution of some particular action (or sequence of actions). We can therefore specify the free energy that is expected to occur at time τ *given* some control state u_t by conditioning all of the distributions in equation 3 on u_t:

$$\mathcal{F}_\tau(\phi_\tau, u_t) = \mathbb{E}_{Q(o_\tau,x_\tau|u_t,\phi_\tau)}[\ln Q(x_\tau|u_t, \phi_\tau) - \ln P(x_\tau, o_\tau|u_t)] \tag{4}$$

This equation defines the expected free energy for time τ given control state u_t, which we denote $\mathbf{G}_\tau(\phi_\tau, u_t)$, i.e. $\mathbf{G}_\tau(\phi_\tau, u_t) = \mathcal{F}_\tau(\phi_\tau, u_t)$. Equation 4 is the form of expected free energy used in the main text.
