# Supplementary material for: Learning action-oriented models through active inference
Source: PLoS Comput Biol. 2020 Apr 23;16(4):e1007805. doi: 10.1371/journal.pcbi.1007805 (PMC7200021; doi:10.1371/journal.pcbi.1007805)
Supplement: S3 Appendix — In this appendix, we decompose expected free energy into instrumental and epistemic value. (PDF) [file pcbi.1007805.s003.pdf]

Appendix 3

In this appendix, we demonstrate that expected free energy is composed of both instrumental (goal-directed) and epistemic (uncertainty-reducing) components. In the main text, we defined expected free energy $\mathbf{G}_\tau(\phi_\tau, u_t)$ as:

$$\mathbf{G}_\tau(\phi_\tau, u_t) = \mathbb{E}_{Q(o_\tau, x_\tau|u_t, \phi_\tau)}[\ln Q(x_\tau|u_\tau, \phi_\tau) - \ln P(x_\tau, o_\tau|u_t)] \tag{1}$$

We can factorize the generative model over future variables as $P(x_\tau, o_\tau|u_t) = P(x_\tau|o_\tau, u_t)P(o_\tau)$, allowing us to rewrite equation 1 as:

$$\mathbf{G}_\tau(\phi_\tau, u_t) = \mathbb{E}_{Q(o_\tau, x_\tau|u_t, \phi_\tau)}[\ln Q(x_\tau|u_t, \phi_\tau) - \ln P(x_\tau|o_\tau, u_t) - \ln P(o_\tau)] \tag{2}$$

This factorization is desirable as it exposes the prior probability of future observations $P(o_\tau)$, a distribution that describes an agents 'preferences' in a manner that is independent of any particular control states u or unknown variables x. However, it additionally exposes the (intractable) posterior distribution $P(x_\tau|o_\tau, u_t)$. The need to evaluate this distribution can be circumvented by noting that it is approximated by the predictive approximate posterior $Q(x_\tau|o_\tau, u_t, \phi_\tau)$ i.e. $Q(x_\tau|o_\tau, u_t, \phi_\tau) \approx P(x_\tau|o_\tau, u_t)$. This is due to the fact that the approximate posterior is implicitly conditioned on the previous history of observations, due to the belief update scheme inherent in the active inference framework.

Applying this approximation to equation 2, we can derive:

$$\begin{aligned} \mathbf{G}_\tau(\phi_\tau, u_t) &\approx \mathbb{E}_{Q(o_\tau, x_\tau|u_t, \phi_\tau)}[\ln Q(x_\tau|u_t, \phi_\tau) - \ln Q(x_\tau|o_\tau, u_t, \phi_\tau) - \ln P(o_\tau)] \\ &= \underbrace{\mathbb{E}_{Q(o_\tau, x_\tau|u_t, \phi_\tau)}\big[\ln Q(x_\tau|u_t, \phi_\tau) - \ln Q(x_\tau|o_\tau, u_t, \phi_\tau)\big]}_{\text{(Negative) epistemic value}} \\ &\quad \underbrace{- \mathbb{E}_{Q(o_\tau, x_\tau|u_t, \phi_\tau)}\big[\ln P(o_\tau)\big]}_{\text{(Negative) instrumental value}} \end{aligned} \tag{3}$$

In the second equality, we have taken the prior probability of observations $P(o)$ into its own expectation in order to clarify the separation between epistemic value (first term) and instrumental value (second term). The second equality of equation 3 is the form of expected free energy used in the main text.
