# Supplementary material for: Learning action-oriented models through active inference
Source: PLoS Comput Biol. 2020 Apr 23;16(4):e1007805. doi: 10.1371/journal.pcbi.1007805 (PMC7200021; doi:10.1371/journal.pcbi.1007805)
Supplement: S4 Appendix — In this appendix, we demonstrate that epistemic value—a component of expected free energy—is equivalent to a number of established formalisms. (PDF) [file pcbi.1007805.s004.pdf]

Appendix 4

In this section we demonstrate that epistemic value - a component of the expected free energy functional - is equivalent to a number of established formalisms. In the main text, (positive) epistemic value was defined as $\mathbb{E}_{Q(o_\tau, x_\tau|u_t)}[\ln Q(x_\tau|o_\tau, u_t) - \ln Q(x_\tau|u_t)]$, an equation which can be rewritten as:

$$
\begin{aligned}
&\mathbb{E}_{Q(o_\tau, x_\tau|u_t)}[\ln Q(x_\tau|o_\tau, u_t) - \ln Q(x_\tau|u_t)] \\
&= \mathbb{E}_{Q(o_\tau|u_t)Q(x_\tau|o_\tau, u_t)}[\ln Q(x_\tau|o_\tau, u_t) - \ln Q(x_\tau|u_t)] \\
&= \mathbb{E}_{Q(o_\tau|u_t)}\Big[\mathbb{E}_{Q(x_\tau|o_\tau, u_t)}[\ln Q(x_\tau|o_\tau, u_t) - \ln Q(x_\tau|u_t)]\Big] \\
&= \mathbb{E}_{Q(o_\tau|u_t)}\Big[\mathbb{KL}[Q(x_\tau|o_\tau, u_t)|| \ln Q(x_\tau|u_t)]\Big]
\end{aligned} \tag{1}
$$

The first equality is derived by factorizing the predictive approximate posterior as $Q(o_\tau, x_\tau|u_t) = Q(o_\tau|u_t)Q(x_\tau|o_\tau, u_t)$, the second equality is derived through the standard property of expectations over random variables, and the final equality is reached through the standard definition of the KL-divergence.

The final equality of equation 1 quantifies the KL-divergence between the agents predictive approximate posterior after taking into observations into account $Q(x_\tau|o_\tau, u_t)$ and before taking observations into account $Q(x_\tau|u_t)$. It is therefore an (expected) KL-divergence between posterior and prior beliefs, a quantity known as (expected) Bayesian surprise. Maximizing Bayesian surprise entails sampling observations that will lead to the greatest change in beliefs, or in other words, sampling observations that will disclose the maximum amount of information.

We can rearrange the final equality of equation 1 as:

$$
\begin{aligned}
&\mathbb{E}_{Q(o_\tau|u_t)}\Big[\mathbb{KL}[Q(x_\tau|o_\tau, u_t)|| \ln Q(x_\tau|u_t)]\Big] \\
&= \mathbb{E}_{Q(o_\tau|u_t)}\Big[\mathbb{E}_{Q(x_\tau|o_\tau, u_t)}[\ln Q(x_\tau|o_\tau, u_t) - \ln Q(x_\tau|u_t)]\Big] \\
&= \mathbb{KL}[Q(x_\tau, o_\tau|u_t)||Q(x_\tau|u_t)Q(o_\tau|u_t)] \\
&= \mathbf{I}(\mathcal{X}_\tau; \mathcal{O}_\tau|u_t)
\end{aligned} \tag{2}
$$

where $\mathbf{I}(\cdot)$ is the mutual information. To reach the final equality we have utilized the fact that the third equality is the definition of mutual information, i.e. $\mathbf{I}(X;Y) = \mathbb{KL}[P(X,Y)||P(X)P(Y)]$. Epistemic value therefore quantifies the mutual information between beliefs and observations, conditioned on control. Mutual information scores the amount of information one gains about a random variable X from observing some other random variable Y. Acting in order to maximize the mutual information between beliefs and observations is equivalent to acting in order to reduce the uncertainty in those beliefs, which can be seen by rewriting equation 2 as:

$$
\mathbf{I}(\mathcal{X}_\tau, \mathcal{O}_\tau|u_t) = \mathbf{H}[Q(x_\tau|u_t)] - \mathbb{E}_{Q(o_\tau|x_\tau, u_t)}\Big[\mathbf{H}[Q(x_\tau|o_\tau, u_t)]\Big] \tag{3}
$$

where $\mathbf{H}[\cdot]$ is the Shannon entropy, a standard measure of uncertainty. Expected free energy will therefore be maximized when observations minimize the entropy (i.e. uncertainty) in the beliefs encoded by $Q(x_\tau)$.
