# Supplementary material for: Learning action-oriented models through active inference
Source: PLoS Comput Biol. 2020 Apr 23;16(4):e1007805. doi: 10.1371/journal.pcbi.1007805 (PMC7200021; doi:10.1371/journal.pcbi.1007805)
Supplement: S5 Appendix — In this appendix, we derive the update equations for beliefs about hidden states, control states and model parameters. (PDF) [file pcbi.1007805.s005.pdf]

Appendix 5

In this section we derive the update equations for (beliefs over) hidden states, control states and model parameters. We first write out the free energy functional in terms of the sufficient statistics and parameters, before finding the derivative of this functional with respect to the sufficient statistics ϕ of the recognition distribution. We then solve these resulting equations for 0 (i.e. where the gradient of free energy is 0 with respect to the sufficient statistics), and rearrange to find the updates for ϕ that minimize free energy. The free energy for the current time point $\mathcal{F}_t(\phi, o_t)$ is given by:

$$\begin{aligned}\mathcal{F}_t(\phi, o_t) &= -\mathbb{E}_{Q(x_t|\phi_t)}[\ln P(o_t|x_t)] + \mathbb{KL}[Q(x_t|\phi_t)||P(x_t)] \\ &= -\mathbb{E}_{Q(s_t|\phi_{s_t})}[\ln P(o_t|s_t, \lambda)] + \mathbb{KL}[Q(s_t|\phi_{s_t})||P(s_t|s_{t-1}, u_{t-1}, \theta)] \\ &\quad + \mathbb{KL}[Q(u_t|\phi_{u_t})||P(u_t)] + \mathbb{KL}[Q(\theta|\phi_\alpha)||P(\theta|\alpha)]\end{aligned} \tag{1}$$

where the first equality was derived in Appendix 1, and the second equality utilizes the factorization of the generative model and recognition distribution. We can rewrite the second equality of equation 1 in terms of parameters of the generative model and sufficient statistics of the recognition distribution:

$$\begin{aligned}\mathcal{F}_t(\phi, o_t) = &\underbrace{\phi_{s_t} \cdot (-\ln \lambda \cdot \vec{o_t})}_{-\mathbb{E}_{Q(s_t|\phi_{s_t})}[\ln P(o_t|s_t,\lambda)]} + \underbrace{\phi_{s_t} \cdot (\ln \phi_{s_t} - \bar{\theta}^{u_{t-1}} \cdot \phi_{s_{t-1}})}_{\mathbb{KL}[Q(s_t|\phi_{s_t})||P(s_t|s_{t-1},u_{t-1},\theta)]} + \\ &\underbrace{\phi_{u_t} \cdot (\ln \phi_{u_t} - (-\vec{\mathbf{G}} \cdot \phi_{u_t}))}_{\mathbb{KL}[Q(u_t|\phi_{u_t})||P(u_t)]} + \\ &\underbrace{\sum_{ij} \left((\phi_{\alpha_{ij}} - \alpha_{ij})\bar{\theta}_{ij} - \ln \Gamma(\phi_{\alpha_{ij}})\right) + \sum_j \ln \Gamma(\phi_{\alpha_j^0})}_{\mathbb{KL}[Q(\theta|\phi_\alpha)||P(\theta|\alpha)]}\end{aligned} \tag{2}$$

where $\phi_{\alpha_j^0} = \sum_k \phi_{\alpha_{kj}}$, $\bar{\theta}_{ij} = \mathbb{E}[\ln \theta_{ij}] = \psi(\phi_{\alpha_{ij}}) - \psi(\phi_{\alpha_j^0})$ and $\vec{o_t}$ is a one-hot encoded vector specifying the current observation. This derivation follows from $\mathbb{E}(x) = \sum_x P(x)x = P \cdot x$ when P is a vector of probabilities for the various values of x held in the vector x. We now collect terms from equation 2 to give:

$$\begin{aligned}\mathcal{F}_t(\phi, o_t) = \phi_{s_t} \cdot (\ln \phi_{s_t} - \ln \lambda \cdot \vec{o_t} - \bar{\theta}^{u_{t-1}} \cdot \phi_{s_{t-1}} + \vec{\mathbf{G}} \cdot \phi_{u_t}) + \phi_{u_t} \cdot \ln \phi_{u_t} + \\ \sum_{ij} \left((\phi_{\alpha_{ij}} - \alpha_{ij})\bar{\theta}_{ij} - \ln \Gamma(\phi_{\alpha_{ij}})\right) + \sum_j \ln \Gamma(\phi_{\alpha_j^0})\end{aligned} \tag{3}$$

We can now differentiate free energy with to the individual sufficient statistics of ϕ, giving:

$$\begin{aligned}\frac{\partial \mathcal{F}(\phi, o)}{\partial \phi_{s_t}} &= \mathbf{1} + \ln \phi_{s_t} - \ln \lambda \cdot \vec{o_t} - \bar{\theta}^{u_{t-1}} \cdot \phi_{s_{t-1}} \\ \frac{\partial \mathcal{F}(\phi, o)}{\partial \phi_{u_t}} &= \mathbf{1} + \ln \phi_{u_t} - \tilde{\mathbf{G}} \\ \frac{\partial \mathcal{F}(\phi, o)}{\partial \phi_\alpha} &= \frac{\partial \bar{\theta}}{\partial \phi_\alpha}(\phi_\alpha - \alpha - \phi_{s_t}\phi_{s_{t-1}})\end{aligned} \tag{4}$$

Where we have ignored the derivative of $\tilde{\mathbf{G}}$ with respect to ϕ_{s_t} for simplicity. Including this would lead to an additional 'optimism bias' term in the first equality. Finally, the variational updates that minimize free energy can be obtained by solving for zero and rearranging, giving:

$$\begin{aligned}\ln \phi_{s_t} &= \ln \lambda \cdot \vec{o_t} + \bar{\theta}^{u_{t-1}} \cdot \phi_{s_t} \\ \ln \phi_{u_t} &= -\tilde{\mathbf{G}} \\ \phi_\alpha &= \alpha + \phi_{s_t}\phi_{s_{t-1}}\end{aligned} \tag{5}$$

To obtain the updates provided in the main text, we apply the softmax operator on $\ln \phi_{s_t}$ and $\ln \phi_{u_t}$, giving the updates for ϕ_{s_t} and ϕ_{u_t}, respectively.
