# Supplementary material for: Learning action-oriented models through active inference
Source: PLoS Comput Biol. 2020 Apr 23;16(4):e1007805. doi: 10.1371/journal.pcbi.1007805 (PMC7200021; doi:10.1371/journal.pcbi.1007805)
Supplement: S6 Appendix — In this appendix, we present results for an additional experiment where we compare learning under epistemic and random action strategies in a high-dimensional state space. (PDF) [file pcbi.1007805.s006.pdf]

# Appendix 6

In simulations presented in the main text, the random and the epistemic agent learn models at a similar rate. We hypothesized that this may have been due to the simplicity of the transition distribution used in the current simulations, meaning that randomly choosing actions gave a near-optimal set of samples to learn from. To test this, we implemented a simple simulation in a larger state space. This simulation was of a 15 x 15 grid world, where agents sense the world directly and simply had to determine the transition dynamics. There were 225 different control states (one for each grid position), and each control state had a corresponding transition distribution, leading to 255 x (15 x 15) = 57375 parameters. Note that this simulation and the corresponding generative model is distinct from the one used in the main text. We investigated the learning of an epistemic and random agent within a single trial (with an altered learning rate of 1). Figure A in S6 Appendix shows the KL-divergence of the transition distributions from the true transition distribution over the course of this trial. It is evident that the epistemic agent learns in a more efficient manner than the random agent. The size of the parameter space means that towards the start of the trial, the random agent searches in a near optimal manner. However, the consistently linear nature of the epistemic agents learning curve demonstrates that the agent searched the state space in a principled manner. This suggests that sampling to reduce epistemic uncertainty provides a principled method for learning a model of environment dynamics.

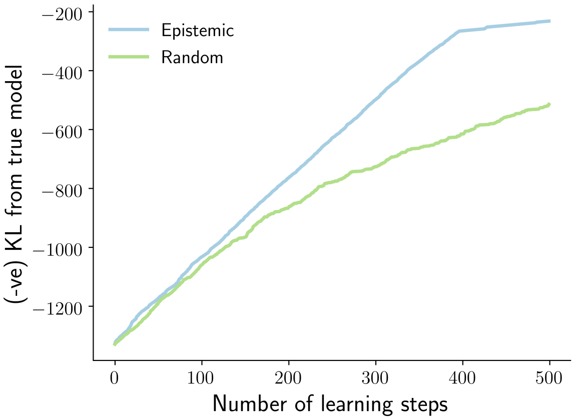


Figure A. **High dimensional model accuracy:** A comparison of the model accuracy of the epistemic and random strategies in a high dimensional state space.
