# Supplementary material for: Learning action-oriented models through active inference
Source: PLoS Comput Biol. 2020 Apr 23;16(4):e1007805. doi: 10.1371/journal.pcbi.1007805 (PMC7200021; doi:10.1371/journal.pcbi.1007805)
Supplement: Attachment — Submitted filename: Response.pdf [file pcbi.1007805.s007.pdf]

Reviewer 1

Major point 1: "*The paper does not report how many control states are used for the simulation (nor the grid size, number of transition functions and parameters). By reading the paper I had the impression that only 2 control states were used. However, Appendix 6 mentions that the simulation is the same as the main text but with a bigger grid (15x15); and in this case 255 control states were used (which is somewhat odd, given that as at any moment the agent can select between run and tumble; is this correct?). Was the same method (one control state for each grid position) used also in the main simulation? And in that case, how did the agent know about its grid position? This point has also some theoretical implications as if so many control states are necessary, the idea that action-oriented models are more parsimonious than models encoding (for example) position is questionable.*"

We agree with the reviewer that this point is both subtle and potentially not clear in the manuscript. Indeed, two control states are used (this is stated on page 13 line 281 and page 34 line 847), along with two hidden states. However, the environment itself is continuous - in the sense that the agent's position, orientation and equations of motion are all continuous. This is noted on page 12 line 266, where we state:

"*Note that while agents operate on discrete representations of the environment, the true states of the environment (i.e the agent's position, the location of the chemical source, and the chemical concentrations) are continuous.*"

In other words, the agents sense a discrete (binary) representation of a continuous variable (the gradient).

We believe the confusion may have arisen from Appendix 6, which was simply meant to be a proof-of-principle demonstration that epistemic value does aid exploration in larger generative models. Here, we used a discrete environment ("This simulation was of a 15 x 15 grid world") entirely distinct from that in the main text. And in this distinct simulation, the agent can choose to transition to any state (hence 255 control states). We have now made it clearer that that the simulation in Appendix 6 is not the same as the one used in the main text, and to further help avoid confusion, we have added the following sentence to Appendix 6:

"Note that this simulation and the corresponding generative model is distinct from the one used in the main text."

Major point 2: "*It would be interesting to discuss (or even better to simulate, but this is fully optional) how well-learned models afford transfer learning. For example, how fast the different agents readapt to changes of prior preferences for gradients. This is important as for many biological organisms preferences can change over time.*"

The reviewer is correct to identify transfer learning as one of the key areas where action-oriented models may inhibit effective learning. One would expect this to be true when the novel behavioural set is not a subset of existing sensorimotor coordination. However, in the case that the new behaviour *is* a subset of existing sensorimotor coordination (i.e. going from walking to running, typing on a keyboard to playing the piano), we would expect action-oriented models to be beneficial, in so far as they reduce the search space for learning the new behaviour. This is closely related to the notion of curriculum learning, where complex behaviours are learned progressively by first learning a series of simpler behaviours. We would tentatively expect that action-oriented models would be useful in those scenarios.

We have included the following passage in the discussion (on the page 30 line 733), with a hope that it facilitates future work:

"It is natural to ask whether there are scenarios in which action-oriented models might impede effective learning and adaptation. One such candidate scenario is transfer learning [1], whereby existing knowledge is reapplied to novel tasks or environments. This form of learning is likely to be important in biology, as for many organisms preferences can change over time. If the novel task or environment requires a pattern of sensorimotor coordination that is distinct from learned patterns of sensorimotor coordination, then a more exhaustive model of the environment might indeed facilitate transfer learning. However, if adaptation in the novel task or environment can be achieved through a subset of existing patterns of sensorimotor coordination (i.e. in going from walking to running), then one might expect an action-oriented representation to facilitate transfer learning, in so far as such representations reduce the search space for learning the new behaviour. This type of transfer learning is closely related to curriculum learning [2], whereby complex behaviours are learned progressively by first learning a series of simpler behaviours. We leave it to future work to explore the scenarios in which action-oriented models enable efficient transfer and curriculum learning."

[1] Lu, Jie, Vahid Behbood, Peng Hao, Hua Zuo, Shan Xue, and Guangquan Zhang. "Transfer learning using computational intelligence: A survey." Knowledge-Based Systems 80 (2015): 14-23.
[2] Bengio, Yoshua, Jérôme Louradour, Ronan Collobert, and Jason Weston. "Curriculum learning." In Proceedings of the 26th annual international conference on machine learning, pp. 41-48. 2009.

Major point 3: *"In the light of the author's discussion about exploration and exploitation, the most useful comparison would be with an "epsilon-greedy instrumental" agent. Is the epsilon-greedy mechanism (which is much simpler than epistemic exploration) sufficient to prevent bad bootstrap in instrumental agents? This could be discussed or (optionally) simulated."*

We agree with the reviewer that, in the current simulations, an epsilon-greedy strategy may be sufficient to overcome bad-bootstraps. We note that the concept of "epsilon-greedy

instrumental” agent might be redundant, as a “pure” epsilon-greedy strategy entails acting instrumentally and occasionally taking random actions. We have included the following line in the discussion (page 27 line 646, following from our existing mention of epsilon-greedy) to highlight this point:

“While an epsilon-greedy strategy might help overcome `bad-bootstraps’ by occasionally promoting exploratory actions, the undirected nature of random exploration is unlikely to scale to complex environments.”

Minor point 1: “*The authors cast the problem as POMDP. However, there is a one-to-one mapping between states and observations. Why not cast it as an MPD?”*

The simulations in the current paper could indeed have been run as an MDP. However, the active inference formalism is explicitly situated in the context of POMDPs. While none of the behaviours would be affected, a number of the equations would change when moving to MDPs, meaning that the paper would become less of a pedagogic aid. The use of identity mappings between states and observations is quite common in the active inference literature when the purpose of the paper is to investigate the transition dynamics implied by active inference (for instance, active inference agents in [1,2,3] directly sense their location through a similar one-to-one mapping mechanism).

[1] Kaplan, Raphael, and Karl J. Friston. "Planning and navigation as active inference." Biological cybernetics 112, no. 4 (2018): 323-343.
[2] Mirza, M. Berk, Rick A. Adams, Christoph D. Mathys, and Karl J. Friston. "Scene construction, visual foraging, and active inference." Frontiers in computational neuroscience 10 (2016): 56.
[3] Friston, Karl J., Richard Rosch, Thomas Parr, Cathy Price, and Howard Bowman. "Deep temporal models and active inference." Neuroscience & Biobehavioral Reviews 90 (2018): 486-501.

Minor point 2: “*The idea of representational inaccuracies is interesting. Is it fair to say that the inaccuracies that emerge in the transition functions are (just) ignorance, deriving from not having sampled some of the (infrequent or unselected) transitions?”*

This is a fair conceptualisation of the representational inaccuracies presented in the manuscript. However, the key argument is that certain sustained patterns of sensorimotor coordination will ensure that these infrequent transitions remain infrequent. We believe that it is a fruitful direction for future work to explore more “explicit” representational inaccuracies.

Minor point 3: “*Page 7, around line 133. Please explain better the difference between control states and actions (and why the former are necessary in active inference but not in other frameworks), as these may sound confusing to most readers.”*

The delineation between control states and actions is for purely formal reasons, and we agree with the reviewer that it might seem to imply something more fundamental in the simulations.

We have changed page 7 line 132 to read:

“The delineation of control states from actions helps highlight the fact that actions are something which occur “in the world”, whereas control states are unknown random variables that the agent must infer”.

Minor point 4: “*Page 11, around line 117. The authors argue that "The goal is not, therefore, to construct a model that accurately captures the true causal structure underlying observations". This is correct, but it may be argued that "the goal is not necessarily" to do so. To the extent that knowing the true causal structure (or a close approximation) is useful, it could be learned by active inference agents.*”

We agree with the reviewer that for some cases, knowing the true causal structure will be beneficial for action. However, we believe this does not change the argument that the *goal* is not to uncover this structure, but instead to enable adaptive agent-environment interactions.

We have changed page 11 line 218 to:

“Therefore, the goal is not necessarily to construct a model that accurately captures the true causal structure underlying observations, but is instead to learn a model that is tailored to a specific set of prior preferences, and thus tailored to a specific set of agent-environment interactions.”

Minor point 5: *“Page 11, around line 238. I think that for the sake of clarity the authors could reiterate that agent (i) is the "sum" of agents (ii) and (iii)”*

We have changed page 11 line 239 read:

“These are the (i) minimization of expected free energy, (ii) maximization of instrumental value, (iii) maximization of epistemic value, and (iv) random action selection, where the minimization of expected free energy (i) corresponds to a combination of the instrumental (ii) and epistemic (iii) strategies.”

Minor point 6: *“Page 12, around line 268. Please explain "spatially rather than temporally"*

We have updated page 12 line 270 to read:

“The chemical gradient is computed as a function of space (whether the agent is facing towards the chemical source) rather than time (whether the agent is moving towards the chemical source) [60], and thus only depends on the agent’s current position and orientation, and the position of the chemical source.”

Minor point 7: "*Figure 2 is interesting and useful. However, it (or its caption) should explicitly mention and explain all the parameters. There are some parameters like lambda and alpha that are not introduced in the caption."*

We thank the reviewer for highlighting this oversight. The caption of figure two has been updated to include the following sentences:

"In this figure, x denotes the variables that an agent infers and φ denotes the parameters of the approximate posterior."

"Here, λ denotes the parameters of likelihood distribution and α denotes the parameters of the prior distribution over parameters. We again refer readers to the methods section for full descriptions of these distributions."

Minor point 8: *"Figure 3 is well explained in the text. Despite so, it risks to be confusing (for distracted readers) as the text mentions that agents can learn for 1000 time steps and the x axis also reports an interval between 0 and 1000 (but it is about learning steps) - hence some readers may conflate the two and believe that the x axis reports time steps for action and not learning. Maybe you could simply change the interval in the x axis?"*

We understand this concern entirely. However, we are unsure how changing the interval will help differentiate the two (the current intervals read 0, 200, 400, 600 - so do not mention the number 1000). We have added the following sentence to the caption of figure three, with the hope that this will help avoid any confusion for the reader:

"Note that the x-axis denotes the number of time steps in the learning phase, rather than the number of time steps in the subsequent testing phase."

Minor point 9: "*The reported results seem to show that the overall performance of (all) agents is not excellent. Could you comment on that?"*

To ensure that we address the reviewers concerns, we highlight two related issues: (i) why does it take agents so long to achieve good performance on the task? And (ii) why is the final distance (i.e after 1000 learning steps) not zero?

As for point (i) - it is a difficult task for two reasons: first, the learning rate is set to a low value, allowing us to simulate learning over time in a simple toy problem, and second, a significant proportion of agents get stuck in the suboptimal behavioural set. The chemotaxis task was intentionally chosen as a problem that contained two "attractive" behavioural sets, where one of these is sub-optimal.

As for point (ii) - the instrumental agent achieves poor final performance as a significant proportion of these agents get stuck in "bad-bootstraps". As for the epistemic, expected free

energy and random agents - this is an artefact of both the chemotaxis strategy and code. First, the distance is measured from the centre of the agent to the centre of the source. Second, the running-tumbling strategy entails a "jittering" around the chemical source, such that it is rare that an agent stops directly on top of the source and keeps tumbling. Instead, it gets extremely close to the source and does a series of running and tumbling behaviours within its (very) local vicinity. If we had made the chemical source not be a single point (i.e a small basin), then we may have found the final distance to converge to zero. We have added the following sentence to page 15 line 355 to explain these aspects of the result:

"The final distance from the source for the expected free energy, epistemic and random agents is not zero due to the nature of the adaptive-hill climbing chemotaxis strategy, which causes agents to not to settle directly on the source, but instead navigate around its local vicinity."

Minor point 10: "*Pag, 16, around line 354. The authors mention that they "measure the accuracy of the expectation of the approximate posterior distribution". Please clarify why you measure the expectation."*

Agent's maintain beliefs about their own beliefs about transition probabilities (i.e. they maintain beliefs about their own generative model). The generative process defines a set of transition probabilities between states but does not define beliefs about these transition probabilities.

We have updated the page 16 line 369 read:

"Due to the fact that the true generative process does not admit the notion of a prior, we measure the accuracy of the expectation of the approximate posterior distribution over parameters θ, i.e. $E[Q(\theta|\varphi\alpha)]$".

Minor point 11: "*The (interesting) results reported in figure 4 suggest that the EFE agent samples neg-neg transitions less often than the instrumental agent. Why this?"*

This is because the instrumental agent is more susceptible to the "bad bootstrap" inherent in our environment, which is the behavioural profile of tumbling in both positive and negative gradients. This behavioural profile is more likely to lead to negative - negative transitions (relative to the alternative, which is tumbling in negative gradients and running in positive gradients).

Minor point 12: "*In Figure 4B-D, it would be useful to show the distribution over states of the "true model" (the same used to measure model accuracy in Figure 4A. This would help making sense of some results; for example, the error of the instrumental agent is lower than the EFE agent; does this imply that the distributions it recovers (e.g., 53) are correct? Note also that these numbers are a bit difficult to interpret given that (as I mentioned above) things like the size of the grid etc. are not mentioned. Adding more details (and perhaps a figure) about the simulation scenario would make the results easier to interpret."*

We thank the reviewer for the helpful comments. We have updated the caption of figure four to help elucidate the meaning of the numbers:

“The numbers indicate the percentage of time that the corresponding state transition was encountered. For instance, the top left box denotes the percentage of time the agent experienced negative to negative state transitions following a tumbling action.”

We agree that adding a panel showing the distribution over states visited by a “true model” would be useful, but we fear it would add additional clutter. Instead, we have added an additional note to the caption of figure four which states that the distribution over states visited by the epistemic and random strategies is (within some very small margin of error) the same as the distributions encountered by a “true model”:

“Note that the distribution of transitions encountered by the epistemic and random strategies corresponds, within a small margin of error, to the distribution of transitions encountered by a `true' model, i.e. a model that has been learned from infinite transitions with no behavioural biases.”

With respect to the interesting point regarding the (active) error of the instrumental agent being lower than the expected free energy agent: this does not mean that the distribution it recovers is correct - indeed, the key point of this figure is to show that “global” model accuracy is not necessarily a predictor of accuracy in the presence of behaviour.

Minor point 13: “*Pag. 21, around line 485. The discussion of model complexity is interesting but could appear counterintuitive. After all, more parameters means more complexity. It may be useful to remark that pruning is necessary in in such kind of models?”*

Indeed, the fact that expected free energy contains the most redundant parameters, combined with the line (486) “Under the assumption that redundant parameters can, and should, be pruned, the expected free energy and instrumental strategies learn simpler models, compared to the epistemic and random strategies”, suggests that these strategies learn simpler models.

We have appended an additional sentence to line 507 page 22, which alludes to the notion that pruning is necessary for action-oriented models:

“These results additionally suggest that pruning parameters will prove to be more beneficial (in terms of model complexity) for action-oriented models.”

Minor point 14: *“Pag 22, around line 519. It would be useful to mention that the agent used in the simulation is the EFE agent?”*

We have updated the page 23 line 539 to read:

“We initialize an agent with a randomly selected maladapted model and allow the agent to interact with (and learn from) the environment according to the expected free energy action strategy (i.e using the E.F.E agent)”

Reviewer 2

Minor point 1: ***“****As an overall comment, some of the figures were very difficult to read (for example 4B). I could follow the narrative in the paper, but in these cases the figures were not very helpful.”*

We agree with the reviewer that figure four, in particular, conveys a large amount of information. We have provided some additional information for the caption of figure four, with the hope that it helps clarify the meaning of the figures:

“The numbers indicate the percentage of time that the corresponding state transition was encountered. For instance, the top left box denotes the percentage of time the agent experienced negative to negative state transitions following a tumbling action.”

Minor point 2: ***“****p.8: The authors rightfully point out that active inference “proposes that an agent’s generative model is biased towards favourable states of affairs”. However, in the current simulation these “prior preferences” are taken as a given. Given that learning takes centre-stage in their paper, and given that the authors take all the other argumentative steps. Perhaps they can say something about how these preferences are acquired (learning seems not to be an option here, especially because of the bad-bootstrapping kind of scenarios the authors point out).”*

We agree entirely with the reviewer that the learning (or acquiring) of prior preferences is one of the most interesting, and underexplored, aspects of the active inference framework. We have included the following sentence on page 8 line 150:

“These prior preferences could be learned from experience, or alternatively, acquired through processes operating on evolutionary timescales.”

Minor point 3: ***“****p.14: The authors introduce 4 learning strategies. At first I thought that the results of the paper were trivial, because, for example, an epistemic agent is explicitly not made to seek out the goal state. But since these strategies are only employed in the learning stage and not in the evaluation stage, this worry was unnecessary. Still, the authors might say something about their expectations in why specifically these models were chosen. And whether the results they obtained were anticipated.”*

We thank the reviewer for this useful suggestion, and for realising that the results were not trivial. With respect to model choices, we have appended the following sentences to page 15 line 321:

"These models were chosen to explore the relative contributions of instrumental and epistemic value to model learning, and crucially, to understand their combined influence. We predict that, when acting to minimize expected free energy, agent's will engage in a form of goal-directed exploration that is biased by their prior preferences, leading to adaptive action-oriented models. In contrast, we expect that (i) the instrumental agent will occasionally become entrenched in bad-bootstraps, due to the lack of exploration, and (ii) the epistemic agent will explore portions of state space irrelevant to behaviour, leading to slower learning."

Minor point 4: "p.17: *"In contrast, the distributions sampled by the expected free energy and instrumental strategies are heavily biased towards a running-induced transition from positive to positive gradients. This is the transition that occurs when an agent is 'running up the chemical gradient', i.e., performing chemotaxis. The bias means that the remaining state transitions are sampled less, relative to the epistemic and random strategies.*

I found it hard to parse these few sentences. Not sure if the "positive" to "positive" is a mistake (or a different meaning of transition is implied here). It might be good to explain the idea of a state transition briefly in the paper (it bears the connotations of state transitions in physics, while what is implied here is a qualitative change in behaviour (i.e. tumbling or running)."

We thank the reviewer for highlighting the lack of clarity in the above paragraph. The agent represents a single discrete hidden state - the chemo-gradient, which can be positive or negative. While the environment is continuous, we can still take a discrete view of the dynamics and identify transitions from positive gradients to negative gradients as a function of time. We understand the concern that this will lead to confusion with different notions of state transitions.

We have first amended line 403 page 18 to read:

"In contrast, the distributions sampled by the expected free energy and instrumental strategies are heavily biased towards a running-induced transitions from positive gradients to again a positive gradient. This is the transition that occurs when an agent is 'running up the chemical gradient', i.e., performing chemotaxis. The bias means that the remaining transitions between states are sampled less, relative to the epistemic and random strategies."

We have also included the following sentence on page 18 line 396:

"Here, the notion of a state transition refers to a change in the state of the environment as a function of time, i.e. a positive to negative state transition implies that the agent was in a positive chemical gradient at time t and a negative chemical gradient at t + 1"

This sentence follows directly from the first mention of “state transitions”, and we hope that it dispels any confusion for the subsequent sections.

Minor point 5: “*p.26: I think the “goal-directed exploration” point, in contrast to more traditional solutions to the explore-exploit trade-off can be highlighted a bit more. Does EFE provide a more principle or integrated solution to this trade-off than more discrete ones (i.e. in which there is an irreversible switch from exploring to exploiting)?”*

We thank the reviewer for the useful suggestion. Indeed, the contrast to discrete solutions to the exploration-exploitation dilemma (which are the paradigmatic approach in RL) provides the perfect context to discuss the implications of goal-directed exploration. We have updated the exploration-exploitation section of the discussion (page 27 line 656) to expand upon these points. The full paragraph now reads (with the new content in italics):

“This is exemplified by approaches that conduct an initial `exploration' phase - in which the task is to construct a veridical model from as few samples as possible - followed by a subsequent `exploitation' phase. By contrast, our approach highlights the importance of `goal-directed exploration', in which the aim is not to resolve all uncertainty to construct a maximally accurate representation of the environment, but is instead to selectively resolve uncertainty until adaptive behaviour is (predicted to be) possible. *Moreover, we have demonstrated that goal-directed exploration allows exploration to be contextualised by an agent’s goals. Specifically, we have shown that acting to simultaneously explore and exploit the environment causes exploration to be biased towards parts of state space that are relevant for goal-directed behaviour, thereby increasing the efficiency of exploration. Therefore, our work suggests that acting to minimise expected free energy can benefit learning by naturally affording an efficient form of goal-directed exploration.*”

Minor point 6: ***“****I think the authors are very well on top of the literature, and I appreciate them engaging with the more theoretical/philosophical literature out there. I think the central idea of the paper can be very well be connected to what Bruineberg, Kiverstein and Rietveld (2016) call the “crooked scientist”. The EFE-agent as 1.) having non-negotiable preferences for the sensation it expects/wishes to encounter and 2) through this, exploring only that part of state-space that is relevant for its goal-directed activities. This second point really comes out nicely in the author’s paper and is a great continuation of the “crooked scientist” line of thinking. Although, the 2015 paper does not discuss expected free-energy, a follow up paper, Bruineberg et al., (2018) does discuss expected free-energy. I think the points made there (in the context of niche construction) are very much in line with and complementary to the current paper.”*

We agree with the reviewer that *Bruineberg et al., (2018)’s* notion of a crooked scientist is the perfect complement to the current work. These existing parallels have been highlighted in the discussion (page 31 line 790):

“The arguments and simulations presented in this paper resonate with previous work which views an active inference agent as a “crooked scientist” [1,2]. Here, an agent is seen as a “scientist” insofar as it seeks out information to enable more accurate predictions. However, this work additionally highlights the fact that agents are biased by their own non-negotiable prior beliefs and preferences, leading them to seek out evidence for these hypotheses. We have built upon this previous work by exploring the types of models that are learned when an agent acts as a “crooked scientist’.”

[1] Bruineberg, Jelle, Julian Kiverstein, and Erik Rietveld. "The anticipating brain is not a scientist: the free-energy principle from an ecological-enactive perspective." Synthese 195, no. 6 (2018): 2417-2444.
[2] Bruineberg, Jelle, Erik Rietveld, Thomas Parr, Leendert van Maanen, and Karl J. Friston. "Free-energy minimization in joint agent-environment systems: A niche construction perspective." Journal of theoretical biology 455 (2018): 161-178.
